# The Impact of Liver Cell Injury on Health-Related Quality of Life in Patients with Chronic Liver Disease

**DOI:** 10.1371/journal.pone.0151200

**Published:** 2016-03-18

**Authors:** Yvonne Alt, Anna Grimm, Liesa Schlegel, Annette Grambihler, Jens M. Kittner, Jörg Wiltink, Peter R. Galle, Marcus A. Wörns, Jörn M. Schattenberg

**Affiliations:** 1 I. Department of Medicine, University Medical Center of the Johannes Gutenberg University, Mainz, Germany; 2 Department of Psychosomatic Medicine and Psychotherapy, University Medical Center of the Johannes Gutenberg University, Mainz, Germany; University of Navarra School of Medicine and Center for Applied Medical Research (CIMA), SPAIN

## Abstract

**Background:**

Patients with chronic liver disease often suffer from unspecific symptoms and report severe impairment in the quality of life. The underlying mechanisms are multifactorial and include disease-specific but also liver related causes. The current analysis evaluated the association of hepatocellular apoptosis in non-viral chronic liver disease and health-related quality of life (HRQL). Furthermore we examined factors, which influence patient's physical and mental well-being.

**Methods:**

A total of 150 patients with non-infectious chronic liver disease were included between January 2014 and June 2015. The German version of the Chronic Liver Disease Questionnaire (CLDQ-D), a liver disease specific instrument to assess HRQL, was employed. Hepatocellular apoptosis was determined by measuring Cytokeratin 18 (CK18, M30 Apoptosense ELISA).

**Results:**

Female gender (5.24 vs. 5.54, p = 0.04), diabetes mellitus type II (4.75 vs. 5.46, p<0.001) and daily drug intake (5.24 vs. 6.01, p = 0.003) were associated with a significant impairment in HRQL. HRQL was not significantly different between the examined liver diseases. Levels of CK18 were the highest in patients with NASH compared to all other disease entities (p<0.001). Interestingly, CK18 exhibited significant correlations with obesity (p<0.001) and hyperlipidemia (p<0.001). In patients with cirrhosis levels of CK18 correlated with the MELD score (r = 0.18, p = 0.03) and were significantly higher compared to patients without existing cirrhosis (265.5 U/l vs. 186.9U/l, p = 0.047). Additionally, CK18 showed a significant correlation with the presence and the degree of hepatic fibrosis (p = 0.003) and inflammation (p<0.001) in liver histology. Finally, there was a small negative association between CLDQ and CK18 (r = -0.16, p = 0.048).

**Conclusion:**

Different parameters are influencing HRQL and CK18 levels in chronic non-viral liver disease and the amount of hepatocellular apoptosis correlates with the impairment in HRQL in chronic non-viral liver diseases. These findings support the role of liver-protective therapies for the improvement of the quality of life in chronic liver disease.

## Introduction

Chronic liver diseases (CLD) are a major cause of morbidity and mortality worldwide. The underlying causes include chronic viral and alcoholic hepatitis, cholestatic, autoimmune and metabolic diseases, all of which can progress to cirrhosis and hepatocellular carcinoma [1]. Patients with chronic liver disease often suffer from unspecific symptoms including fatigue, muscle and joint pain, loss of appetite, digestive problems, anxiety, depression and other emotional problems, which can profoundly decrease their quality of life and well-being [2]. Health-related quality of life (HRQL) as a multidimensional concept includes subjective evaluations of domains related to physical, emotional, mental and social functioning in a context of a disease and its treatment [3]. Several studies showed significantly reduced HRQL in patients with CLD compared to healthy controls [4–6]. In advanced chronic liver disease cirrhosis and its complications such as ascites, spontaneous bacterial peritonitis, hepatic encephalopathy and recurrent variceal bleeding are associated with severe impairments in health-related quality of life [1, 7]. The severity of disease, as determined by stage of fibrosis or Child-Turcotte-Pugh (CTP) scores, but also risk factors leading to chronic liver disease, seem to have a considerable relationship with HRQL [8]. To assess liver injury and therefore the severity and degree of fibrosis in liver diseases, liver biopsy represents the gold standard [9]. This invasive procedure is associated with possible complications and due to sampling errors the biopsy may not completely represent the stage of liver disease [10]. To overcome these diagnostic uncertainties, non-invasive markers of hepatocellular injury and inflammation have been developed. Cytokeratin (CK) 18, an intermediary filament protein expressed by hepatocytes, is proteolytically cleaved during hepatocellular apoptosis. The emerging CK18 fragments are released into the bloodstream and can be detected with the monoclonal antibody M30 by an enzyme-linked immunosorbent assay (ELISA) [11]. Several studies have shown increased serum levels of CK18 in acute and chronic liver diseases but not in healthy individuals [11–13]. Cytokeratin 18 therefore reflects hepatocellular apoptosis and can be used as marker for non-invasive diagnosis of acute and chronic liver disease [14]. The aim of the study was to evaluate the impact of hepatocellular injury as determined by CK18 levels on the reported health-related quality of life in different non-infectious chronic liver diseases.

## Materials and Methods

### Patient characteristics

Patients with non-infectious chronic liver disease who were referred to the outpatient clinic of the University Medical Centre of the Johannes Gutenberg University Mainz between January 2014 and June 2015 were included after informed consent was given and analyses were performed retrospectively. Exclusion criteria were malignant disease, sepsis, and previous liver transplantation, myocardial infarction within the last six months or participation in other therapeutic trials.

The etiology of liver disease was determined clinically and following liver histology. Liver cirrhosis was diagnosed based on histological features, laparoscopy, ultrasound or clinical signs including ascites, hepatic encephalopathy, thrombocytopenia, splenomegaly, and laboratory findings indicative of impaired liver function. Liver histology was considered if performed within 12 month of attendance. Additionally, transient elastography (Fibroscan®) was performed at the time of attendance or within three month of the outpatient visit. In patients with NAFLD or NASH fibrosis was determined by calculating the NAFLD fibrosis score if biopsy and transient elastography were missing [15]. Diabetes mellitus, hypertension, hyperlipidemia and the metabolic syndrome were defined according to the definitions of the Joint Scientific Statement for Harmonizing the Metabolic Syndrome [16]. Individual medication was recorded and body mass index (BMI) was calculated by measurement of height and weight (BMI = weight (kg) / height^2^ (m^2^)). Obesity was defined as BMI ≥ 30 kg/m^2^. Laboratory results were obtained at the time of attendance and were considered missing if not available within a maximum of 30 days.

### Ethical consideration

The study was approved by the Ethikkommision der Landesärztekammer Rehinland-Pfalz. All clinical investigation have been conducted according to the principles expressed in the Declaration of Helsinki. Written informed consent was obtained from the participants.

### Cytokeratin 18 measurement

CK18 was determined in serum following centrifugation of whole blood at 2000 r/min for 10 min and storage of the serum at −80°C for a maximum of 12 months. Serum CK18 levels were quantified by commercially available M30 Apoptosense® ELISA kit (PEVIVA, Bromma, Sweden) according to the manufacturer's instructions. CK18 levels >200 U/l were considered elevated according to published data (14) and the manufacturer’s report [17].

### Questionnaire

For health-related quality of life (HRQL) the liver disease-specific questionnaire “Chronic Liver Disease Questionnaire (CLDQ)” in the German version was used [18, 19]. The CLDQ-D consists of 29 items on a seven-point Likert scale ranging from 1 (all of the time) to 7 (none of the time) representing the frequency of clinical symptoms and emotional problems associated with CLD in the last two weeks. It is divided into six subscale scores (abdominal symptoms, fatigue, systemic symptoms, activity, emotional functioning, worry) and a CLDQ-D overall score. By dividing each domain score by the number of items in the domain, CLDQ-D results can be presented on a 1–7 scale with 1 indicating worst HRQL and 7 indicating best HRQL. Patients were asked to complete the questionnaire during regular outpatient visit, on the same day blood was taken for analyzing CK18.

### Statistical analysis

Descriptive statistics were computed for all variables. These include means and standard deviations or medians and 25^th^ and 75^th^ percentiles for continuous factors. For categorical variables, frequencies and percentages were calculated. Univariable regression analysis was used to examine association between two variables. Differences between groups were calculated by Mann-Whitney-U-rank test or the Kruskal-Wallis rank test. All tests were two-tailed, with a significant P value defined as <0.05. All data were analyzed using IBM SPSS Statistic Version 21.0 (Armonk, NY: IBM Corp.).

## Results

### Patient characteristics

**Fig 1:**
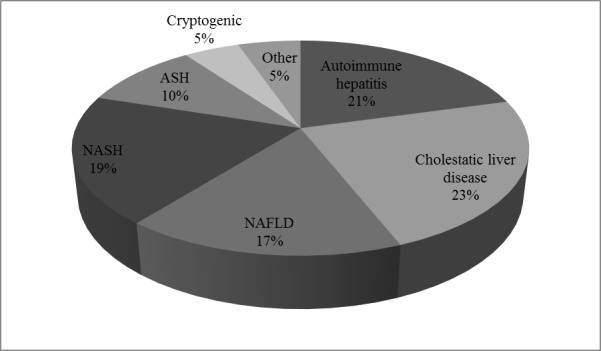
Etiologies of the underlying chronic liver disease.

A total of 150 patients with non-infectious chronic liver disease were included between January 2014 and June 2015. The mean age for the study population was 52 years (range 22–77), of which 64.7% (n = 97) were female. Cholestatic liver disease was the most frequent underlying non-infectious chronic liver disease in 23.3%, including PBC, PSC and overlap syndrome, followed by autoimmune hepatitis in 20.7%. NAFLD and NASH were present in 16.7% and 19.3%, whereas in 10% alcohol-induced liver disease was present. Other liver diseases including drug induced hepatopathy, haemochromatosis, alpha-1 antitrypsin deficiency and Budd-Chiari-syndrome were found in 5.3%. In 4.7% the underlying CLD was cryptogenic. Etiologies of the underlying chronic liver disease are pictured in Fig 1. Biopsy was available in 54% (n = 81). Demographic data, prevalence of metabolic risk factors, complications and characteristics of liver function in relation to underlying chronic liver disease are presented in Table 1.

**Table 1:** Demographic data, prevalence of metabolic risk factors, complications and characteristics of liver function in relation to underlying chronic liver disease.

Characteristic		Cholestatic liver disease (n = 35)	Autoimmune hepatitis (n = 31)	NASH (n = 29)	NAFLD (n = 25)	ASH (n = 15)	Cryptogenic (n = 7)	Other (n = 8)
Male gender *		9 (25.7)	3 (9.7)	14 (48.3)	12 (48)	11 (73.3)	2 (28.6)	2 (25.0)
Age ^+^		55 (32; 76)	54 (23; 75)	52 (24; 76)	50 (22; 69)	64 (40; 77)	42 (29; 57)	52 (32; 76)
Obesity *		5 (14.3)	8 (25.8)	20 (69.0)	13 (52.0)	2 (13.3)	0 (0)	3 (37.5)
Type II diabetes *		5 (14.3)	3 (9.7)	8 (27.6)	2 (8.0)	5 (33.3)	0 (0)	0 (0)
Hypertension *		10 (28.6)	12 (38.7)	14 (48.3)	9 (36.0)	12 (80.0)	0 (0)	2 (25.0)
Hyperlipidemia *		8 (22.9)	8 (25.8)	20 (69.0)	24 (96.0)	3 (20.0)	4 (57.1)	3 (37.5)
Cirrhosis *		7 (20.0)	5 (16.1)	10 (34.5)	1 (4.0)	15 (100.0)	0 (0)	3 (37.5)
CTP-Score*								
	A	6 (17.1)	3 (9.7)	9 (31.0)	0 (0)	4 (26.7)	0 (0)	2 (25.0)
	B	1 (2.9)	1 (3.2)	1 (3.4)	1 (4.0)	9 (60.0)	0 (0)	1 (12.5)
	C	0 (0)	1 (3.2)	0 (0)	0 (0)	2 (13.3)	0 (0)	0 (0)
ALT ^#^		28 (21; 40)	28 (18; 45)	49 (38; 72)	43 (33;65)	23 (18; 41)	51 (5; 109)	33 (25; 42)
AST ^#^		31 (28; 35)	28 (24; 34)	43 (32; 50)	33 (30; 39)	38 (31; 61)	37 (24; 47)	35 (27; 49)
Bilirubin ^#^		0.7 (0.5; 0.8)	0,6 (0.5; 1.0)	0.8 (0.5; 1.2)	0.7 (0.4; 0.9)	1.4 (0.8; 2.1)	0.5 (0.4; 0.8)	0.6 (0.5; 0.9)
INR ^#^		0.9 (0.9; 1.0)	1.0 (0.9; 1.0)	1.0 (0.9; 1.0)	1.0 (0.9; 1.0)	1.1 (1.0; 1.3)	1.0 (0.9; 1.1)	1.0 (0.9; 1.1)
Creatinine ^#^		0.8 (0.72; 0.85)	0.8 (0.7; 0.9)	0.9 (0.8; 0.9)	0.8 (0.7; 0.9)	1.0 (0.8; 1.1)	0.7 (0.7; 0.8)	0.8 (0.8; 1.0)
Albumin ^#^		38 (36; 40)	38 (35; 40)	40 (37; 42)	41 (40; 42)	31 (24; 35)	70 (16; 113)	39 (35; 42)
Platelet count ^#^		261 (228; 323)	236 (200; 302)	221 (163; 266)	238 (215; 281)	147 (90; 179)	254 (207; 349)	219 (142; 285)
MELD score ^#^		4.6 (3.9; 5.5)	4.8 (4.2; 6.0)	5.1 (4.0; 6.2)	4.7 (3.7; 6.2)	7.5 (6.5; 9.6)	3.9 (3.5; 5.1)	4.9 (3.8; 6.9)

ALT (normal range -<50 U/l), AST (normal range 5–35 U/l), Bilirubin (normal range <1 mg/dL), Creatinine (normal range 0.6–1.1mg/dL), Albumin (normal range 34–48 g/l), platelet count (normal range 150-450/nL); Obesity is defined as BMI >30kg/m^2^

*Data presented in (n [%])

^+^Data presented in (median [range]

^#^Data presented in (median [interquartile range])

### CLDQ in chronic non-viral liver disease

The mean CLDQ-overall score was 5.35 (±1.1) in the patient population. The lowest scores were reported in the subcategory "fatigue" with a value of 4.61 (±1.5), followed by "emotional functioning" 5.30 (±1.2), “systematic symptoms” with 5.45 (±1.2) and “worry” with 5.52 (±1.4). “Abdominal symptoms” and “activity” showed relatively higher values with 5.59 (±1.4) and 5.91 (±1.2). With regards to the underlying CLD patients with cholestatic liver disease showed the lowest CLDQ-D overall score, while patients with cryptogenic CLD reported present the highest score. Etiologies and mean CLDQ-D overall score in relation to the underlying CLD are summarized in Table 2.

**Table 2:** CLDQ-D overall score and CK18 levels in relation to the aetiology of chronic liver disease.

CLD	N (%)	CLDQ-D overall score Mean (±SD)	CK-18 level Median (IQR)
All CLD	150 (100)	5.35 (±1.1)	206.0 (107.0; 378.8).
Autoimmune hepatitis	31 (20.7)	5.28 (±1.2)	139.9 (86.8, 276.6)
Cholestatic liver disease	35 (23.3)	5.21 (±1.2)	117.9 (92.9, 192.9)
NAFLD	25 (16.7)	5.57 (±0,8)	247.0 (202.4, 410.7)
NASH	29 (19.3)	5.28 (±1.1)	475.7 (266.0, 605.9)
ASH	15 (10.0)	5.31 (±0.9)	221.4 (132.1, 436.1)
Cryptogenic	7 (4.7)	5.86 (±0.4)	101.1 (87.6, 147.5)
Other	8 (5.3)	5.39 (±1.1)	181.0 (108.9, 305.4)

IQR: interquartile range

Women showed reduced CLDQ-D overall score in comparison to men (mean (SD) 5.24 (±1.0) vs. 5.54 (±1.1), p = 0.04). Especially in the subcategory “systematic symptoms” women exhibited significantly lower scores (p = 0.01). No correlation existed between CLDQ-D overall score and age or BMI. Patients with type 2 diabetes mellitus (15.3%) exhibited a significant reduced CLDQ-D overall score (4.75 (±1.0) vs. 5.46 (±1.0), p<0.001), while the presence of arterial hypertension and hyperlipidemia showed no influence on health-related quality of life. Medication intake, a surrogate of existing comorbidities, significantly influenced HRQL (5.24 vs. 6.01, p = 0.003). Even a prescription of one to three drugs per day (50% of patients) reduced the CLDQ-D overall score in comparison to patients with no medication (13%) (Mean (SD) 5.4 (± 1.0) vs. 6.01 (± 0.7), p = 0.02). Regarding standard laboratory results, no correlations were observed with liver enzymes, platelet counts, albumin, INR or bilirubin. The presence of liver cirrhosis also influenced the reported HRQL. The mean CLDQ-D overall score in patients with liver cirrhosis, which accounted for 27.3% of all patients, was lower compared to patients without cirrhosis (Mean (SD) 5.19 (±1.2) vs. 5.41 (±1.0)), although not reaching statistical significance (p = 0.39). There was no correlation found between CTP or MELD score and CLDQ-D overall score. We observed a reduced overall scores in patients with advanced fibrosis (>F2; n = 48) in contrast to low-grade or no fibrosis (n = 102) (Mean (SD) 5.10 (±1.2) vs. 5.47 (±0.95), p = 0.13). Similarly, patients with high-grade inflammation on liver biopsy (n = 23) exhibited a reduction of the CLDQ-D overall scores in comparison to patients with low-grade or no inflammation (n = 58; mean (SD) 4.93 (±1.1) vs. 5.32 (±1.1), p = 0.096).

### Determination of hepatocellular apoptosis in CLD

Cytokeratin 18 (CK18) fragments in the entire cohort were at a median of 206 U/l (IQR 107.0; 378.8). Results of CK18 levels in non-infectious chronic liver disease in relation to patient characteristics and laboratory results are summarized in Table 3.

**Table 3:** CK18 levels in non-infectious chronic liver disease in relation to patient characteristics and laboratory results.

Characteristics	CK18 level (U/l)		
	Yes	No	p
Male gender ^#^	261.1 (132.1; 439.5)	186.9 (101.1; 314.2)	**0.047**
Obesity ^#^	333.7 (186.9; 529.8)	164.3 (100.0; 298.7)	**<0.001**
Type II diabetes ^#^	252.4 (128.6; 508.9)	195.2 (103.6; 376.1)	0.190
Hypertension ^#^	202.4 (114.3; 369.5)	215.0 (101.1; 404.1)	0.881
Hyperlipidemia ^#^	254.0 (147.5; 475.7)	150.0 (92.9; 273.4)	**<0.001**
Cirrhosis ^#^	265.5 (138.2; 400.4)	186.9 (101.1; 344.0)	**0.047**
Advanced Fibrosis ^#^	389.4 (252.4; 529.8)	201.6 (103.6; 378.8)	**0.004**
High-grade inflammation ^#^	529.8 (389.4; 667.1)	178.6 (103.6; 265.5)	**<0.001**
	**Correlation coefficient (r)**		**p**
ALT	0.48		**<0.001**
AST	0.47		**<0.001**
γ-GT	0.25		**0.002**
Bilirubin	0.18		**0.027**
INR	0.13		0.11
Creatinine	0.02		0.85
Albumin	0.09		0.29
Platelet count	-0.12		0.13
CRP	0.32		**0.002**
Ferritin	0.24		0.06
MELD score	0.18		**0.03**
CLDQ-D	-0.16		**0.048**

^#^ Data presented in (median [interquartile range]); Obesity is defined as BMI >30kg/m^2^; advanced Fibrosis is defined as F3 or F4; γ-GT = γ-glutamyltransferase. To compare different CK18 levels Mann-Whitney-U-rank test was performed. Statistical dependence between laboratory levels, MELD score, CLDQ-D-score and CK18 levels was measured by Spearman's rank correlation coefficient.

Interestingly, BMI influenced serum CK18 significantly as patients with higher BMI exhibited higher values of CK18 (r = 0.29, p<0.001) and obesity correlated with CK18 levels (p<0.001) across all disease entities. The frequency of obesity in the different etiologies of liver disease is display in Table 4. No association was observed with regards to age, the presence of diabetes type 2 or arterial hypertension, while hyperlipidemia was also associated with significantly higher CK18 levels (p<0.001). Highest scores were observed in patients with NASH (Median (IQR) 475.7 U/l (266.0; 605.9), while cryptogenic liver disease showed the lowest scores ((Median (IQR) 101.1 U/l (87.6; 147.5), which was statistically significant (p<0.001). Etiologies and mean CK18 values with regard to the underlying CLD are summarized in Table 2. In patients with liver cirrhosis CK18 levels were significantly higher than in patients without underlying cirrhosis, although levels of CK18 did not discriminate between the CTP categories. However, CK18 levels differed significantly with regards to the presence or absence of advanced fibrosis (p = 0.004) and the highest CK18 levels were observed in advanced fibrosis. Furthermore the presence of inflammation influenced the amount of CK18. Patients with histologically-defined high-grade inflammation (n = 23) exhibited significantly higher serum CK18 levels in comparison to patients with low-grade inflammation (n = 49; median (IQR) 529.8 (389.4; 667.1) vs. 178.6 (103.6; 265.5), p<0.001). In univariate variance analysis a significant association between inflammation and CK18, independently of the underlying disease, was observed (p<0.001). With regards to standard laboratory results, a positive correlation of CK18 and aspartate aminotransferase (AST) (r = 0.47, p<0.001) and alanine aminotransferase (ALT) (r = 0.48, p<0.001) was observed (Table 3). Patients with higher MELD scores and therefore reduced liver function showed significantly elevated CK18 (r = 0.18, p = 0.03).

**Table 4:** Frequency of obesity in the study population with chronic liver disease.

CLD	Obesity (n = 51)	% of all obese patients
Autoimmune hepatitis	8	15.7
Cholestatic liver disease	5	9.8
NASH/NAFLD	33	64.7
ASH	2	3.9
Cryptogenic	0	0
Other	3	5.9

Women exhibited lower levels of CK18 compared to men (p = 0.047). In subgroup analysis there were no gender specific differences in age, BMI, presence of type 2 diabetes mellitus, arterial hypertension or hyperlipidemia. Regarding fibrosis stage, women presented with a lower grade or no fibrosis more often (p = 0.027). Also, Child-Turcotte-Pugh (CTP) was significantly different (p = 0.026), as 76% of women had no cirrhosis in comparison to 66% of men. CTP stage C was not present in women.

### Role of hepatocellular apoptosis on the reported quality of life

To asses a potential influence of the degree of hepatocellular apoptosis on the HRQL, CLDQ-D and CK18 were correlated. There was a significant negative correlation of CK18 and the health-related quality of life (r = -0.16, p = 0.048). The analysis of the subcategories of the CLDQ showed a significant correlations between “worry” (r = -0.21, p = 0.01) and "fatigue" (r = -0.17, p = 0.04) with CK18. No correlation was observed between CK18 and "abdominal symptoms", "systemic symptoms", "activity" and "emotional functioning".

### Non-cirrhotic liver disease

In a subgroup analysis we examined patients without cirrhosis (n = 109). In this subpopulation there was no significant correlation between CK18 and QOL (r = -0.136, p = 0.16), only in the category "fatigue" a negative, significant correlation (r = -0.214, p = 0.025) was observed. CK18 was associated with the stage of fibrosis (Median (IQR) high grade: 464.6 (178.6; 552.7) vs. low grade: 172.4 (100.0; 311.9); p = 0.005) and the amount of inflammation (Median (IQR) high grade 565.3 (475.7; 667.1) vs. low grade: 140.2 (92.7; 244.8); p<0.001). In this subpopulation there were also significant correlations between CK18 and BMI (r = 0.364, p<0.01), obesity (p<0.001) and hyperlipidemia (p<0.001).

### Non-NASH/NAFLD patients

96 patients without NASH/NAFLD were analyzed. In this subgroup an association between CK18 and CLDQ (r = -0.219; p = 0.032) was observed. CK18 was associated with the histological stage of fibrosis (p<0.001) and the amount of inflammation (p = 0.005). In this subpopulation there were no correlations between CK18 and BMI (r = 0.02, p = 0.86), obesity (p = 0.160) or hyperlipidemia (p = 0.987). Regarding laboratory findings, higher AST (r = 0.4, p<0.001), ALT (r = 0.32; p = 0.002) and MELD (r = 0.27; p = 0.009) were associated with higher CK18 levels.

## Discussion

Chronic liver disease exerts considerable negative impact on the patient’s health-related quality of life [4–6]. In the current study we used the German version of the Chronic Liver Disease Questionnaire (CLDQ) as a validated liver disease-specific instrument for determining HRQL [20]. So far, there have been only few studies to examine HRQL in chronic, non-viral liver disease and most studies were performed in patients with chronic hepatitis C or in patients with advanced liver disease [5]. In a large German cohort with 524 patients suffering from advanced CLD on the transplantation list mean CLDQ-overall score was 4.62 (±1.2) [18]. Jara and colleagues detected an overall score of 5.04 (±1.1) in their study performed on 142 German patients evaluated for liver transplantation [21]. An overall score of 4.69 (±1.3) was found in the study of Hauser et al. with 203 patients suffering CLD in a referral hospital [19]. In our cohort, the overall score was 5.35 (±1.1), representing a higher perceived HRQL in comparison to these other German studies. The most likely reasons are the exclusion of patients with viral hepatitis, the outpatient setting and the lower rate of cirrhosis in the currently studied cohort in patients with early liver disease not on the transplant list. In the current analysis, the most frequently reported symptom was "fatigue" which is comparable to the previous studies [18, 19, 21]. In assessing the role of gender on HRQL, our data show that female patients with CLD had more severe HRQL impairment than male patients. These findings are similar to the study of Afendy and colleagues [8] but were not replicated in other studies on the influence of gender and HRQL [21–23]. Medical treatment and therefore comorbidities are associated with an impaired HRQL. Even a drug intake of one to three drugs per day already reduced HRQL in our cohort. Previous investigations showed similar results [5, 24]. The presence of type 2 diabetes mellitus influences HRQL in patients with chronic liver diseases. This is in concordance with studies that demonstrated a reduced HRQL in patients with type 2 diabetes mellitus in the general population [25, 26]. No association was found with arterial hypertension, hyperlipidemia and obesity. Considering the cause of liver disease, patients with cholestatic liver disease showed the lowest overall scores and patients with cryptogenic CLD or NAFLD presented the highest score, although data did not reach statistical significance. Underlying cause of liver disease does not influence the HRQL [5, 24]. However, HRQL appeared to be dependent on disease severity and an inverse correlation between HRQL and CPT has been reported [2, 21, 24]. It has also been shown that CLDQ-D reliably detects differences in HRQL between non-cirrhotic and patients classified as CTP stage C, but not between single CTP classes [19]. In our cohort we observed a reduction in CLDQ-D overall score in patients with liver cirrhosis in comparison to patients without cirrhosis, although the results did not reach statistical significance. In addition there was no difference between HRQL and CTP score. Considering the small number of patients with cirrhosis (n = 41) in the current analysis, with 24 presenting with CTP stage A, 14 CTP stage B and only 3 patients with CTP stage C, the failure to validity of this result is limited. Furthermore it should be taken into consideration that all patients attended in an outpatient setting, which may have a strong influence in the perceived HRQL. Parameters of liver function showed no direct impact on patients' quality of life, which is similar to the findings by Jara et al. [21]. Furthermore we found no correlation regarding MELD Score, which is also reported by several previous studies [8, 22, 27]. In contrast to the CTP score an increasing MELD does not seem to be related to quality of life since encephalopathy and ascites are factors that contribute to CTP score, but not to MELD.

Cytokeratin 18 is known as a cell death biomarker, which reflects the degree of hepatocellular apoptosis and can be used as non-invasive marker in acute and chronic liver disease [14]. Several studies have analyzed CK18 in different chronic liver disease and healthy controls, including chronic viral hepatitis [14, 28], autoimmune hepatitis and cholestatic liver diseases [12], as well as NAFLD and NASH [11, 29]. CK18 has been extensively studied as a biomarker of NASH and has been shown to be able to distinguish simple steatohepatitis from hepatic steatosis [30, 31]. Of all patients with CLD, patients with NASH exhibited the highest CK18 levels in our cohort. Also, CK18 differentiated between NAFLD and NASH. In the current analysis a positive correlation of CK18, BMI and obesity was demonstrated. The subgroup of patients with non-NAFLD liver diseases (n = 96) exhibited no correlation of CK18 and obesity. However, a high percentage (64.7%) of obese patients was suffering from NASH/NAFLD (Table 4).

In non-NAFLD liver diseases, only 18 patients were obese. This subgroup was too small to be further analyzed for an association of obesity and CK18 and will have to be studied in future trials. Interestingly, women had significant lower CK18 values compared to men. While there were no gender specific differences in metabolic factors, we found differences regarding the disease stage. Women presented with lower grades or no fibrosis more often and CTP was significantly different, as 76% of women had no cirrhosis in comparison to 66% of men. Additionally, CTP stage C was not observed in women. This was likely related to the underlying liver disease. In the study population women suffered more often from cholestatic liver diseases–a total of 74% were female—and autoimmune hepatitis, with 90% being female. On the contrary, men presented more frequently with alcoholic liver disease–in this group, 73% were male. On univariate variance analysis the underlying disease, but not gender influenced CK18.

With regards to liver function, transaminases and γ-glutamyltransferase showed an association with CK18 levels, as previously described [11, 32]. CK18 differentiates between the presence of hepatic cirrhosis, but not CTP classes. Yagmur et al. reported similar results and confirmed our observation that MELD and CK18 are positively correlated [33]. In accordance with previous studies, CK18 was a marker for the presence of liver fibrosis in our cohort [14, 34]. There was a positive correlation between histologically graded inflammation and CK18, as previously described [35].

Interestingly, subgroup analysis of non-cirrhotic participants showed no correlation between CK18 and HRQL. This is likely related to additional complications in cirrhosis, e.g. portal hypertension that can impair the HRQL. In patients without cirrhosis, CK18 was associated with the stage of fibrosis and the amount of inflammation as well as BMI, obesity and hyperlipidemia. This may be influenced by the underlying liver disease, since 40% of non-cirrhotic patients suffered from NASH/NAFLD. In the non-cirrhotic study population, a high proportion of liver disease was related to NASH/NAFLD, which exhibited an association of liver damage (CK-18) and comorbidities, e.g. obesity and hyperlipidemia, but did not show a correlation of CLDQ and CK18. The existing discrepancy of high levels of liver injury and but lack of symptoms and impairment in the quality of life in non-cirrhotic NASH/NAFLD patients puts these patients at particular risk to progress to more advanced disease stages unnoticed. Additionally, the adherence to recommendations concerning life-style changes, medications or screening procedures are potentially lower compared to symptomatic patients.

In the current analysis we observed a weak, negative correlation between Cytokeratin 18 levels and HRQL, reflecting that the degree of hepatocellular apoptosis in patients with chronic non-viral liver disease–among other factors–impairs the quality of life. Previous studies have demonstrated that the severity of disease, determined by stage of fibrosis or CPT scores, is correlated with the HRQL [8]. It remains to be determined if hepatic inflammation and cytokines are the responsible for the loss of quality of life. However, reduction of hepatocellular injury has to be considered a key treatment goal, to achieve an improvement of health–even beyond liver related outcomes.

## Supporting Information

S1 Dataset The impact of liver cell injury on health-related quality of life in patients with chronic liver disease. Gender: 0 = male, 1 = female; age [years]; disease entity: 1 = cryptogenic liver disease, 2 = NASH, 3 = alcohol-induced liver disease (ASH), 4 = autoimmune hepatitis, 5 = primary biliary cirrhosis (PBC), 6 = primary sclerosing cholangitis (PSC), 7 = wilsons disease, 8 = haemochromatosis, 9 = drug induced liver injury, 10 = any overlap syndrome, 11 = NAFLD, 12 = Budd-Chiari-syndrome, 13 = alpha-1 antitrypsin deficiency; M30: CK-18 fragments; DM: type 2 diabetes: 0 = no, 1 = yes; HPT: hypertension: 0 = no, 1 = yes; HPL: hyperlipidemia: 0 = no, 1 = yes; alcohol consumption:0 = no, 1 = < 20g per day, 2 = ≥ 20g per day; height [meter]; weight [kg]; body weight category: 1 = BMI < 20.0kg/m^2^, 2 = BMI = 20.0–24.9kg/m^2^, 3 = BMI = 25.0–29.9kg/m^2^, 4 = BMI > 30.0kg/m^2^; Obesity: BMI > 30kg/m^2^: 1 = no, 2 = yes; histology: 0 = not available, 1 = available; fibrosis: 1 = no fibrosis or low grade fibrosis, 2 = advanced fibrosis (F3-F4); cirrhosis: 0 = no, 1 = yes; CTP classes: Child-Turcotte-Pugh classes: 0 = no cirrhosis, 1 = CTP A, 2 = CTP B, 3 = CTP C; CTP score: Child-Turcotte-Pugh score: value from 5 to 15 points; inflammation: histological inflammation: 1 = no inflammation or low grade inflammation, 2 = advanced inflammation; MELD score; AST: aspartate aminotransferase [U/l]; ALT: alanine aminotransferase [U/l]; γ-GT: Gamma-glutamyl-transferase [U/l]; AP: alkaline phosphatase [U/l]; GLDH: glutamate dehydrogenase [U/l]; albumin [g/l]; Quick [%]; INR; bilirubin [mg/dL]; creatinine [mg/dL]; platelet count [/nL]; HbA1c: glycated hemoglobin [%]; CRP: C-reactive protein [mg/L]; ferritin [ng/dl]; TG: triglyceride [mg/dl]; Cholesterol [mg/dl]; HDL: high-density lipoprotein cholesterol [mg/dl]; LDL: low-density lipoprotein cholesterol [mg/dl]; LDL/HDL: quotient of LDL to HDL [%]; CLDQ_AS: CLDQ_abdominal symptoms; CLDQ_FA: CLDQ_fatigue; CLDQ_SS: CLDQ_systemic symptoms; CLDQ_AC: CLDQ_activity; CLDQ_EF: CLDQ_ emotional functioning; CLDQ_WO: CLDQ_worry; CLDQ_G: CLDQ_overall score; Med: number of daily drug count; Med_category: 0 = no medication, 1 = 1–3 drugs/day, 2 = 4–5 drugs/day, 3 = >5 drugs /day, #NULL! = no data.
(XLSX)
